# Anesthetic experience does not reduce accidental dural puncture in surgical patients: a retrospective case-controlled study

**DOI:** 10.1186/s12871-022-01657-x

**Published:** 2022-05-10

**Authors:** Yosuke Nakadate, Emi Nakajima, Kodai Ikemoto, Takeshi Oguchi, Takashi Matsukawa

**Affiliations:** 1 Department of Anesthesiology, Faculty of Medicine, University of Yamanashi, 1110 Shimokato, Chuo, Yamanashi, 409-3898 Japan; 2 Department of Anesthesiology, University of Tsukuba Hospital, 2-1-1 Amakubo, Tsukuba, Ibaraki 305-8576 Japan

**Keywords:** Accidental dural puncture, Epidural analgesia, Incident reporting system, Risk factor, Surgical patients

## Abstract

**Background:**

Accidental dural puncture (ADP), which is a complication of epidural anesthesia, still exists and leads to worse outcomes in surgical patients. While residency training is important for epidural competency, it remains unknown whether anesthetic experience reduces ADP in surgical patients. Using an incident reporting system along with anesthetic records, this case-controlled study retrospectively investigated risk factors associated with ADP in surgical patients.

**Methods:**

Patients who experienced ADP during epidural anesthesia who were registered in the incident reporting system of our institution between April 2012 and March 2019 were enrolled. Patients with ADP were control-matched with those who without ADP in a 1:3 ratio, to compare the potential risk factors and calculated odds ratios (ORs) for ADP. The primary hypothesis was that anesthesiologists’ experience reduces the incidence of ADP. The secondary hypothesis was that there are risk factors for ADP. Between-group differences in anesthesiologists’ experience were compared using the Mann–Whitney U test. Significance was set at *P* < 0.05.

**Results:**

Thirty-five patients who experienced ADP were identified from the incident reporting system. These were matched with 69 patients who did not experience ADP. There was no difference in the years of experience of anesthesiologists between the groups that did and did not experience ADP (8 [3–20] vs. 9 [3–18] years, respectively; *P* = 0.65).

**Conclusions:**

Having an experienced anesthesiologist did not guarantee the prevention of ADP. Daily individual training and briefings would be needed to reduce the incidence of ADP.

## Background

Epidural anesthesia is a common method of providing postoperative analgesia [1]. Accidental dural puncture (ADP) refers to an anesthesia-related complication [2] and could result in postdural puncture headache [3], prolonged hospitalization, increased medical costs, and delayed rehabilitation [2]. To prevent ADP, we need to recognize identified risk factors: multiple attempts [4], depth to the epidural space [5], and patient movement [4] in labor, old age [6, 7], and lower lumbar approaches [6, 7], in surgical patients. In addition, considering the report that trainees require approximately 50 attempts to achieve epidural competence [8], it would be important for anesthesiologists to be trained in residency programs. However, it is still unclear whether anesthesia residency training is enough for reducing the incidence of ADP in surgical patients.

We, therefore, hypothesized that clinical anesthesiology experience reduces the incidence of ADP in surgical patients. We investigated the potential risk factors using incident reports and anesthetic records. The incident reporting system, which has recently been established to assess and prevent potential health complications [9], is a powerful tool to review each adverse incident in detail. We also discuss the preventive methods for ADP.

## Methods

Our study was approved by the hospital’s Ethics Committee on August 22, 2019 (protocol no. 2105). It followed the recommended Strengthening the Reporting of Observational Studies in Epidemiology (STROBE) guidelines Informed consent was obtained Opt-out on the web-site of Department of Anesthesia, University of Yamanashi. Those who rejected were excluded. This was approved by the ethic board. All methods in the study were conducted in accordance with all requirements outlined by the Ethic Committee and the ethical standards laid down in the amended 1964 Declaration of Helsinki.

### Study design

This study used a retrospective, case-control design to assess the patient- and anesthesiologist-related risk factors for ADP in surgical patients.

### Patient population

We recruited patients with ADP from April 2012 to March 2019 using the incident reports system of our institute. All patients who experienced ADP during epidural anesthesia who were registered in the incident reporting system were enrolled. Patients who opted out were excluded.

It is mandatory to submit these reports whenever an anesthesia-related ADP occurs in our hospital. Two authors extracted and browsed all anesthesia-related reports and identified those that included the phrase “accidental dural puncture.”

The electronic incident reporting system used for this study is operated by the University Hospital. Any healthcare provider can access the platform without restrictions and submit a report. These reports provide a description of the incident cases.

Following the selection of ADP incidents, we obtained data on the patients’ background. We also collected relevant information from the anesthesia and medical records.

In addition, we conducted a retrospective search for all the surgical patients who underwent epidural anesthesia or combined spinal-epidural anesthesia during the same period. We matched each ADP patient with their non-ADP counterpart in a 1:3 ratio to assess the risk factors. We have described the matched factors in Statistical analysis section.

### Data collection

We collected data on age, sex, height, body weight, body mass index, comorbidities, medications, diagnosis, surgical procedure, and the American Society of Anesthesiologists physical status (ASA-PS). In addition, we obtained information on the experience of the anesthesiologist, the number of epidural puncture attempts, number of epidural anesthesia providers, approached intervertebral level, method of approach (median/paramedian), and depth to the epidural space, from the anesthesia records. We also collected additional information that described the event from the incident reports, if available. Missing data were excluded from the analysis.

### Epidural anesthesia and accidental dural puncture

All procedures related to epidural anesthesia were performed in a lateral decubitus position using a 17-gauge Tuohy needle (B Braun Medical Inc., Tokyo, Japan) before anesthetic induction. Each anesthesia provider chose the method of approach—median or paramedian—and approached the intervertebral level. They detected the epidural space using the “loss-of-resistance” technique. After placement of the epidural catheter, we injected 3 mL of 2% mepivacaine as a test dose. The anesthetic dermatomal levels achieved were determined using a cold test. Each attending anesthesiologist defined the occurrence of ADP based on cerebrospinal fluid discharge, unexpected blood pressure drops, and unexpected motor block.

### Hypotheses

The primary hypothesis was that the experience of the anesthesiologist reduces the incidence of ADP in surgical patients. The secondary hypothesis was that there are potential risk factors for ADP.

### Sample size calculation

We calculated the sample size using G*Power (version 3.1) (University of Düsseldorf, 2020) [10]. According to the “G*power” calculation, a sample of 32 patients with ADP and 64 patients without ADP in each group would be required to have a large effect (*d* = 0.50), with α = 0.05, β = 0.2, and patients ratio = 1:2, using the Wilcoxson–Mann–Whitney test.

We estimated that 32 patients should be identified from the incident report for the past 8 years based on the incidence of ADP (0.5%) [6, 7] and the number of patients who underwent epidural anesthesia each year in our hospital (n≒800): 6400 cases of epidural anesthesia in the past 8 years.

### Statistical analysis

Data are presented as mean ± standard deviation or median (interquartile range) for continuous variables and frequencies (percentages) for categorical variables.

We matched the cases and controls in a 1:3 ratio based on the type of surgery (gynecologic, obstetric, upper/lower abdominal intestinal, urological, or thoracic), sex, age (within 10 years), height (within 10 cm), weight (within 10 kg), year of surgery (within 1 year), ASA-PS (within 1 point), and emergency status, considering 30% of unmatched pairs. Patients who experienced ADP were matched with those who did not experience ADP, based on characteristics such as age, sex, weight, and depth to the epidural space, to exclude patient-related factors discussed previously [5–7]. In instances where patients who experienced ADP were matched with fewer than three patients who did not experience ADP, we did not recruit additional patients who did not experience ADP.

Continuous variables were compared between the two groups using Student’s *t*-test or Mann–Whitney *U* test. Frequencies were compared between the two groups using the chi-squared test or Fisher’s exact test.

Logistic regression analysis was used to estimate unadjusted and adjusted odds ratios with 95 confidence intervals for ADP in surgical patients. Experience of anesthesiologist and variables with *P* < 0.05 were included in the regression model, which was adjusted for potential anesthetic confounders (anesthesiologists’ experience, number of epidural puncture attempts, and number of epidural anesthesia providers). Statistical analyses were performed using SPSS (version 23) (IBM Japan, Tokyo, Japan). Significance was set at *P* < 0.05.

## Results

Thirty-five patients with ADP were identified from the incident reporting system. All incident reports were submitted by the anesthesiologist. All incidents occurred in the operating room. The details of the procedure were unavailable (i.e., the step of needle insertion or timing of syringe usage to detect epidural and others).

One doctor (senior resident) reported four incidents of ADP, two doctors reported 3 incidences of ADP each, five doctors reported two incidences of ADP each, and 15 doctors reported one incidence of ADP each.

During the study period, 54 anesthesiologists (staff or senior residents) worked at our hospital. None of the patients who experienced ADP were excluded because no one opted out.

**Fig. 1:**
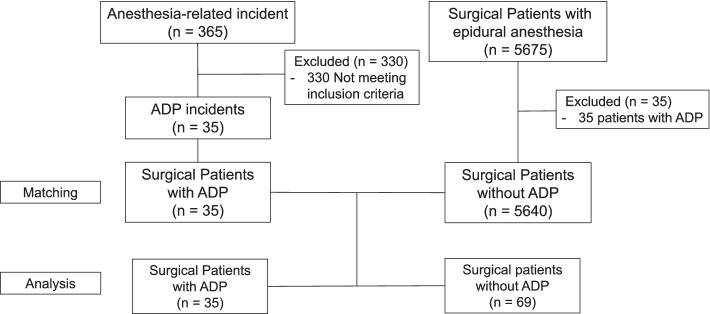
Flow diagram

We also identified 5675 patients who underwent epidural anesthesia during the study period from the anesthesia records. We selected 69 patients without ADP (Fig. 1), and they were control-matched based on their characteristics (Table 1). Eleven patients who experienced ADP were each matched with three patients (controls) who did not experience ADP, 12 patients were each matched with two controls, and a further 12 patients were each matched with one control.

**Table 1:** Patient characteristics

	Patients with ADP (*n* = 35)	Patients without ADP (*n* = 69)	*P*-value
Matched variables			
Age (years)	55 ± 17	55 ± 17	0.92
Sex (male:female)	13:22	27:42	1.00
Height (cm)	160 ± 8	160 ± 8	0.90
Weight (kg)	58 ± 9	58 ± 9	0.98
BMI (kg/m^2^)	23 ± 3	23 ± 4	1.00
ASA-PS	2 [2-2]	2 [2-2]	0.46
Emergency	5 (14)	6 (8)	1.00
Procedures			
Gynecologic	9 (26)	18 (26)	
Caesarean section	8 (23)	16 (23)	
Upper abdominal	6 (17)	11 (16)	
Lower abdominal	6 (17)	13 (19)	
Urologic	5 (14)	8 (16)	
Thoracic	1 (3)	3 (4)	
Non-matched			
Smoking	12 (34)	30 (43)	0.40
Hypertension	13 (37)	22 (32)	0.66
Ischemic Heart disease	3 (9)	9 (13)	0.75
Liver dysfunction	5 (14)	15 (22)	0.44
Kidney dysfunction	4 (11)	6 (9)	0.73
Diabetes	2 (6)	9 (13)	0.33
Dyslipidemia	3 (9)	12 (17)	0.38
Endocrine disease	7 (20)	3 (4)	0.03
Psychiatric disorder	6 (17)	6 (9)	0.21
Foreigner	4 (11)	1 (1)	0.04

Data are presented as mean ± SD, median [interquartile range], or number of patients (%). Some patients had more than one comorbidities

*ADP* Accidental dural puncture, *BMI* Body mass index, *ASA-PS* American Society of Anesthesiologists physical status

Regarding patient characteristics, a higher number of patients with ADP had an endocrine disease (*P* = 0.03) and were foreigners (*P* = 0.04) (Table 1) compared to the controls. The endocrine disorders included Graves’ disease (*n* = 2), hypothyroidism (*n* = 3), adenomatous goiter (*n* = 1), and pituitary dysfunction (*n* = 1). Of the foreign patients, three were Asian and one was South American.

**Fig. 2:**
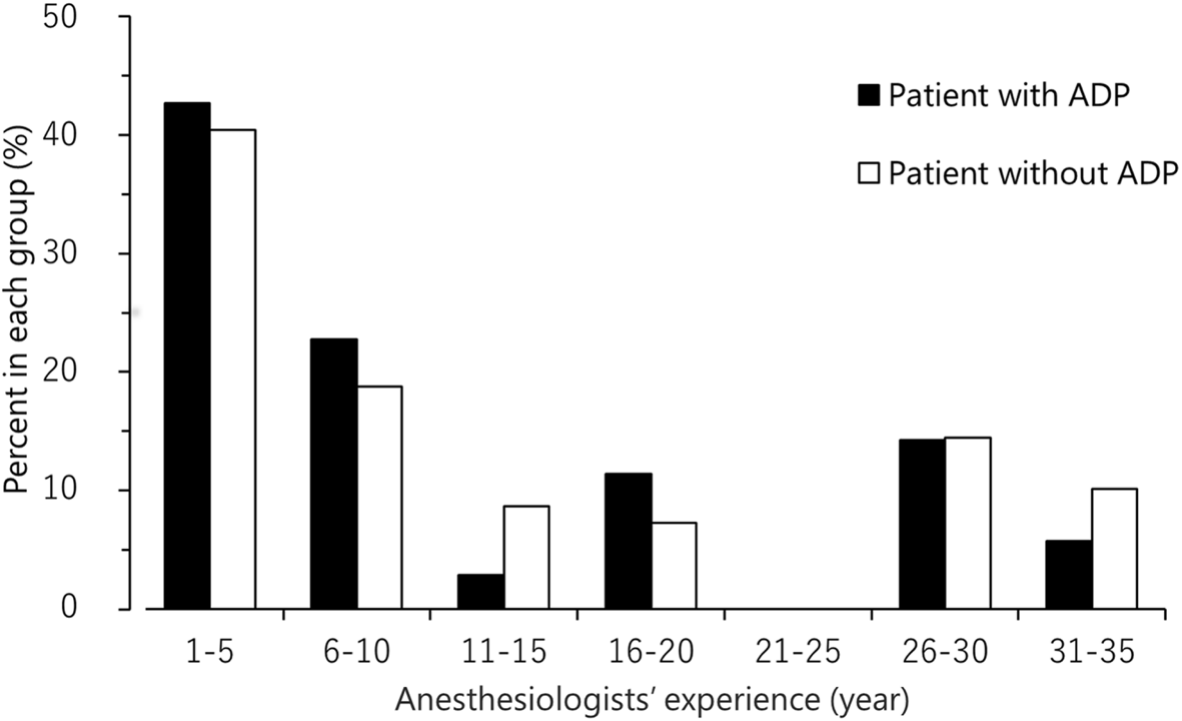
Distribution of the anesthetic experience of epidural anesthesia providers

There was no difference in the years of experience of the anesthesiologists between the groups (patients with ADP vs. patients without ADP: 8 [3–20] vs. 9 [3–18] years, respectively; *P* = 0.65) (Table 2 and Fig. 2). The number of epidural puncture attempts was higher among patients with ADP compared to those without ADP (Table 2). The anesthesia provider was replaced after several trials in three cases in each group.

**Table 2:** Anesthetic factors of epidural anesthesia

	Patients with ADP (*n* = 35)	Patients without ADP (*n* = 69)	*P*-value
Intervertebral level for puncture	T10 [T5–T12]	T10 [T6–T11]	0.17
T4–6	1 (3)	3 (4)	
T7–9	5 (14)	13 (19)	
T10–12	24 (69)	51 (74)	
L1	5 (14)	2 (3)	
Method of approach (median:para-median)	11:21	35:34	0.14
Number of attempts	2 [1, 2]	1 [1–1.8]	< 0.01
Number of operators	1 [1]	1 [1]	0.31
Depth (cm)	4.5 [4.0–5.0]	4.5 [4.0–5.0]	0.30
Experience of the anesthesiologist (years)	8 [3–20]	9 [3–18]	0.65

Data are presented as median [interquartile range] or number of patients (%). The methods of approach in the three patients were unknown

*ADP* Accidental dural puncture

Logistic regression analysis revealed that foreign patients, the presence of endocrine diseases and the number of epidural puncture attempts were independent risk factors for ADP (Table 3).

**Table 3:** Logistic regression analysis of accidental dural puncture

	OR	[95% CI]	*P*-value	Adjusted OR^a^	[95% CI]	*P*-value
Endocrine diseases	5.50	[1.33–22.8]	0.02	5.18	[1.51–26.4]	0.02
Foreign patient	8.77	[0.94–81.7]	0.06	12.1	[1.16–127.0]	0.04
Experience of the anesthesiologist (years)	0.99	[0.94–1.03]	0.70	0.99	[0.94–1.03]	0.71
Number of attempts	1.83	[1.11–3.04]	0.02	1.98	[1.10–3.56]	0.02

Adjusted OR: OR adjusted for the number of epidural puncture attempts and the experience of the anesthesiologist. *Abbreviations*: *CI* Confidential interval, *OR* Odds ratio

## Discussion

In this study, while multiple epidural puncture attempts were identified as a risk factor for ADP, having a more experienced anesthesiologist did not reduce the incidence of ADP in surgical patients. Additionally, foreign patients and endocrine diseases were identified as new patient-related risk factors.

Van de Velde et al. [11] reported that staff anesthesiologists (0.28%) did not reduce the incidence of ADP compared to residents (0.33%) in 55 witnessed ADPs out of 17,198 neuraxial blocks in labor. A case-controlled study reported by Michaan et al. [4] also did not find a difference in anesthesiologists’ experience (mean: cases vs. control: 10.9 vs. 12.4 years, respectively) in 49 cases of blood patches out of 17,977 epidural anesthesia cases in labor. The experience of the anesthesiologist in our study too did not influence the incidence of ADP in surgical patients, despite the greater variability in puncture sites, age, and anatomical changes compared to those of laboring patients. This means neither residency training nor longer experience reduces the incidence of ADP in surgical patients, as well as in labor.

However, the present study did not reveal whether ADP was repeatedly caused by specific anesthesiologists or all anesthesiologists equally. The maximum number of ADPs produced by one individual was only four, while the median number of years of experience as an anesthesia provider in the ADP group was eight. Therefore, more patients with ADP over a longer period are needed to determine the significance of anesthesiologists’ experience. Furthermore, detailed information on the epidural procedure from anesthetic charts and incident reports is needed to analyze individual technique.

In our hospital, while residents are trained under the supervision of the attending anesthesiologist, the attending anesthesiologists themselves have few opportunities to review their own skills and technique. Since anesthesiologists with over 20 years of experience can also cause ADP, repeated and periodical reviewing procedures are needed to maintain and improve their skills.

The key to successful epidural catheter insertion includes the following: skills of the anesthesiologist [8]; patient factors, such as anatomical variations [12] or deformities [13]; and patient positioning during the procedure.

In surgical patients, as well as in labor, multiple epidural attempts are an independent risk factor for ADP. Especially in instances where epidural anesthesia has failed, we should be cautious to prevent ADP. Maintaining optimal positioning [14] and preventing patient movement [4] would play important roles.

Optimal patient positioning is essential for successful epidural placement. Regardless of the position (i.e., sitting, lateral decubitus, jack-knife, or prone position) selected for the initiation of neuraxial procedures, it is useful to have an assistant in front of the patient; this would facilitate attaining maximal spinal flexion [14]. We identified being foreigner as a patient-related risk factor for ADP. Not all foreigners are familiar with the local language. The presence of a language barrier impedes communication. In order to ensure proper communication between the patient and the anesthesiologist during the epidural anesthesia procedure [14], a trained helper and/or interpreter should be present to help relieve anxiety and achieve better communication [15]. An assistant can relieve anxiety by guiding the patient and facilitating position maintenance without moving until the procedure is complete [16].

Endocrine diseases were identified as another patient-related risk factor for ADP. These factors may be related to patients’ movement because there are no reports on the association between the anatomy of the spine and thyroid disease or race. In our hospital, it is not mandatory for patients’ movement to be documented in the anesthetic chart. Further studies are needed to clarify the relationship between endocrine disorders and anxiety and patients’ movement during epidural anesthesia.

Our study has several limitations. First, the patient sample size was small because of the low incidence of ADP. It might affect secondary hypothesis. Therefore, a type-1 error cannot be excluded. Second, it was based on the incident reporting system. Underreporting might have occurred because of the voluntary submission of the reports [17]. A type-2 error cannot be excluded. Third, our analysis did not include a detailed description of the event. Therefore, the incident reporting system needs to be modified for ADP analysis to include a more detailed description of the event. Fourth, epidural anesthesia was performed by few third- and fourth-year anesthetists. This can be attributed to the fact that they were employed at other hospitals during our residency program. Finally, these results might not be applicable to other institutions. In labor, epidural or spinal anesthesia was performed only in caesarean section. Most of the parturients were full-term births because our hospital is not a perinatal medical center. Our department does not have an obstetric section and does not use epidural or spinal anesthesia during vaginal delivery.

Our data suggest that continuous daily anesthetic training is needed. We should also remain cautious in situations where epidural catheter placement has failed in the first attempt, to avoid ADP in surgical patients.

## Conclusions

Greater anesthesiologist experience did not affect the incidence of ADP. Thus, daily individual training and briefing are needed to reduce the incidence of ADP. Endocrine disorders and foreign patients might be added to risk factors for ADP. In order to analyze the relationship between each anesthesiologist’s skill and ADP, details of each epidural anesthesia procedure need to be described in daily anesthetic records and the incident reporting system.

## Data Availability

The datasets used and/or analysed during the current study are available from the corresponding author on reasonable request.

## Abbreviations

ADP: Accidental dural puncture
STROBE: Strengthening the Reporting of Observational Studies in Epidemiology
ASA-PS: American Society of Anesthesiologists physical status
CI: Confidence interval
OR: Odds ratio

## References

[1] Block BM, Liu SS, Rowlingson AJ, Cowan AR, Cowan JA, Wu CL (2003). Efficacy of postoperative epidural analgesia: a meta-analysis. JAMA..

[2] Rana K, Jenkins S, Rana M (2018). Insertion of an intrathecal catheter following a recognised accidental dural puncture reduces the need for an epidural blood patch in parturients: an Australian retrospective study. Int J Obstet Anesth.

[3] Apfel CC, Saxena A, Cakmakkaya OS, Gaiser R, George E, Radke O (2010). Prevention of postdural puncture headache after accidental dural puncture: a quantitative systematic review. Br J Anaesth.

[4] Michaan N, Lotan M, Galiner M, Amzalag S, Many A (2016). Risk factors for accidental dural puncture during epidural anesthesia for laboring women. J Matern Fetal Neonatal Med.

[5] Hollister N, Todd C, Ball S, Thorp-Jones D, Coghill J (2012). Minimising the risk of accidental dural puncture with epidural analgesia for labour: a retrospective review of risk factors. Int J Obstet Anesth.

[6] Meyer-Bender A, Kern A, Pollwein B, Crispin A, Lang PM (2012). Incidence and predictors of immediate complications following perioperative non-obstetric epidural punctures. BMC Anesthesiol.

[7] Kuroda K, Miyoshi H, Kato T, Nakamura R, Yasuda T, Oshita K (2015). Factors related to accidental dural puncture in epidural anesthesia patients. J Clin Anesth.

[8] Drake EJ, Coghill J, Sneyd JR (2015). Defining competence in obstetric epidural anaesthesia for inexperienced trainees. Br J Anaesth.

[9] Staender S (2011). Incident reporting in anaesthesiology. Best Pract Res Clin Anaesthesiol.

[10] Faul F, Erdfelder E, Lang AG, Buchner A (2007). G*power 3: a flexible statistical power analysis program for the social, behavioral, and biomedical sciences. Behav Res Methods.

[11] Van de Velde M, Schepers R, Berends N, Vandermeersch E, De Buck F (2008). Ten years of experience with accidental dural puncture and post-dural puncture headache in a tertiary obstetric anaesthesia department. Int J Obstet Anesth.

[12] Uyl N, de Jonge E, Uyl-de Groot C, van der Marel C, Duvekot J (2019). Difficult epidural placement in obese and non-obese pregnant women: a systematic review and meta-analysis. Int J Obstet Anesth.

[13] Sprung J, Bourke DL, Grass J, Hammel J, Mascha E, Thomas P (1999). Predicting the difficult neuraxial block: a prospective study. Anesth Analg.

[14] El MR, Fleisher L, Wiener-Kronish J, Cohen N, Young W (2014). Miller's Anesthesia.

[15] Gillespie BM, Chaboyer W, Murray P (2010). Enhancing communication in surgery through team training interventions: a systematic literature review. AORN J.

[16] Jones AR, Carle C, Columb M (2013). Effect of table tilt on ligamentum flavum length measured using ultrasonography in pregnant women*. Anaesthesia..

[17] Runciman W, Hibbert P, Thomson R, Van Der Schaaf T, Sherman H, Lewalle P (2009). Towards an international classification for patient safety: key concepts and terms. Int J Qual Health Care.

